# XBot2D: towards a robotics hybrid cloud architecture for field robotics

**DOI:** 10.3389/frobt.2023.1168694

**Published:** 2023-10-04

**Authors:** Luca Muratore, Nikos Tsagarakis

**Affiliations:** Humanoids and Human Centered Mechatronics (HHCM), Istituto Italiano di Tecnologia, Genova, Italy

**Keywords:** cloud robotics, field robotics, unstructured environments, resource allocation, service provisioning, hybrid cloud computing

## Abstract

Nowadays, robotics applications requiring the execution of complex tasks in real-world scenarios are still facing many challenges related to highly unstructured and dynamic environments in domains such as emergency response and search and rescue where robots have to operate for prolonged periods trading off computational performance with increased power autonomy and *vice versa*. In particular, there is a crucial need for robots capable of adapting to such settings while at the same time providing robustness and extended power autonomy. A possible approach to overcome the conflicting demand of a computational performing system with the need for long power autonomy is represented by cloud robotics, which can boost the computational capabilities of the robot while reducing the energy consumption by exploiting the offload of resources to the cloud. Nevertheless, the communication constraint due to limited bandwidth, latency, and connectivity, typical of field robotics, makes cloud-enabled robotics solutions challenging to deploy in real-world applications. In this context, we designed and realized the XBot2D software architecture, which provides a hybrid cloud manager capable of dynamically and seamlessly allocating robotics skills to perform a distributed computation based on the current network condition and the required latency, and computational/energy resources of the robot in use. The proposed framework leverage on the two dimensions, i.e., 2D (local and cloud), in a transparent way for the user, providing support for Real-Time (RT) skills execution on the local robot, as well as machine learning and A.I. resources on the cloud with the possibility to automatically relocate the above based on the required performances and communication quality. XBot2D implementation and its functionalities are presented and validated in realistic tasks involving the CENTAURO robot and the Amazon Web Service Elastic Computing Cloud (AWS EC2) infrastructure with different network conditions.

## 1 Introduction and state-of-the-art

In the past decade, the robotics community has shown great interest in addressing the challenge of the increasing computational demand of robotic systems in order to take advantage of the newly introduced machine learning, perception, online planning, and optimization tools, toward more autonomous robotic machines. Efforts in dealing with this challenge resulted in the cloud computing paradigm and the development of the concept of cloud robotics (Inaba, 1993; Hu et al., 2012). In this context, robots can leverage the cloud to access advanced computational resources, such as data storage, processing power, and Machine Learning (ML)/Artificial intelligence (AI) algorithms. By offloading some of the computational tasks to the cloud, robots can preserve their local resources and improve their performance, and efficiency in terms of reduced energy consumption and reliability (Rahman et al., 2019).

**FIGURE 1:**
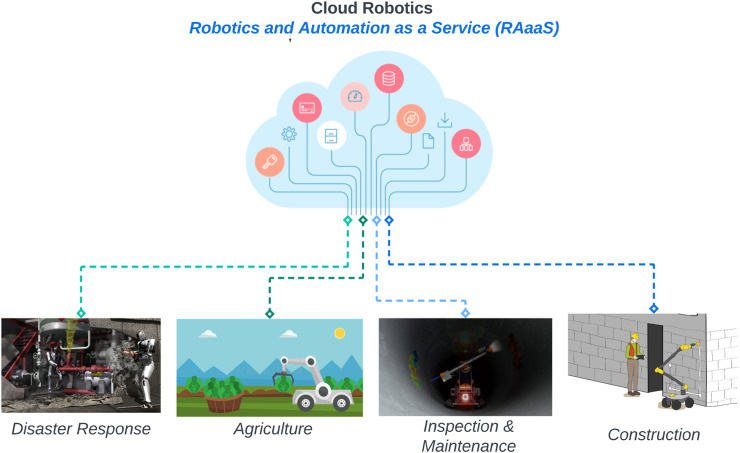
Cloud robotics with the robotics and automation as a service (*RAaaS*) model for application in field robotics, such as disaster response (image copyright DARPA), agriculture, inspection and maintenance (image copyright OMS), and construction (image copyright CONCERT H2020 EU project).

In 2015 Kehoe et al. (2015), introduced the notion of Robotics and Automation as a Service (*RAaaS*), which can be seen as the equivalent model of the Software-as-a-service (*SaaS*) in the robotics field. In *RAaaS*, the software modules are stored on a central cloud server and provided to the robots over the internet (Figure 1): this can offer several benefits for field robotics applications, such as scalability, remote monitoring, improved performance for computational-intensive tasks, and cost-effectiveness.

However, the robotic systems and the cloud data centers are typically multi-hop distance apart, and this causes longer communication time and data transfer delays. As a result, cloud robotics often becomes less suitable for latency-sensitive operation (Afrin et al., 2021), especially for applications in unstructured environments, such as agriculture, construction, disaster response, and inspection and maintenance, where communication represents a significant limitation to the actual deployment of cloud-enabled robots.

The main challenges of the above rely on:

• the limited bandwidth available in such scenarios, which can prevent the transmission of high-resolution sensor data,
• the limited connectivity in remote or isolated field environments, which can restrict the ability of the robot to communicate with the cloud,
• the significant latency in communication due to distance or network congestion, which can prevent the robot from reacting to unexpected changes in the environment,
• the possible electromagnetic interference, which can lead to packet loss.

Given these constraints, the dynamic allocation of resources between the local and the cloud infrastructure became an essential aspect of the *RAaaS*, as it allows the robot to balance its local and remote computing resources and optimize its performance based on the demands of the task to execute and the remaining operational time needed for the mission, as explained in the survey by Afrin et al. (2021). Dynamic resource relocation in *RAaaS* involves moving computational resources between the cloud and the local infrastructure in response to changes in the performance metrics and requirements of the robotics system executing the task. For example, during periods of high computational demand, the robot can offload compute-intensive tasks to the cloud, freeing up local resources for other tasks. Conversely, during periods of low computational demand, the robot can conserve local resources by using cloud resources for tasks that require significant computational power.

The robot’s local computational unit(s) can handle the execution of robotic applications with hard Real-Time (RT) constraints: this is beneficial for latency-sensitive tasks that must ensure predictable response time. However, the onboard computing components can quickly become overwhelmed due to the robots’ size, shape, power supply, motion mode, and working environment (Hu et al., 2012). Moreover, another main challenge in this regard is related to the software architecture employed locally, which must support this kind of RT execution without introducing overhead in the execution. On the other hand, robotic applications use cloud instances to perform large-scale computations [as in Chen et al. (2021)]. Cloud service providers virtualize computing servers and offer various computing instances, including virtual machines (VMs) and containers, to the robotic system. Additionally, cloud instances can be dynamically configured according to the resource requirements of the applications. To summarize, although cloud resources offer higher computational power, they include an additional delay for data transfer, which is unsuitable for latency-sensitive tasks. Furthermore, while robots are capable of supporting hard real-time tasks, they have energy constraints, and their usage in field robotics scenarios can lead to communication issues preventing communication and data transfer to and from the cloud.

Our work focuses on the software architecture design and implementation of a real-world employable hybrid local-cloud framework for a single robotic system. We are not considering the Edge infrastructure and the Fog computing paradigms introduced in the last few years (Mouradian et al., 2018a) in order to have a layer between the robot and the cloud, which will be one of our future works in this context.

The literature on cloud robotics software architecture often relies on ROS (Quigley et al., 2009) and its rapidly growing replacement ROS2 (Macenski et al., 2022). These are open-source middlewares that represent the *de facto* standard for robotics in academia. The main feature of ROS and ROS2 is to support code reuse in robotics research and development: they are a distributed framework of processes that enables executables to be individually designed and loosely coupled at runtime.

Rapyuta, presented by Mohanarajah et al. (2015a), is an open-source platform allowing robots to move their processing to commercial data centers, realizing the cloud robotics paradigm. Robots do not have to perform heavy processing onboard, providing computational environments that are customizable and secure within the cloud. These computing environments enable easy access to the RoboEarth knowledge repository (Waibel et al., 2011). In Rapyuta, robot nodes or Docker images are built on the cloud and pushed to the registered robots. A similar approach is taken by AWS Greengrass (Kurniawan, 2018). Using proprietary interfaces, as reported in Ichnowski et al. (2022), Rapyuta and Greengrass allow building and deploying an entire pipeline for robotics applications, as in Mohanarajah et al. (2015b), Mouradian et al. (2018b), from a centralized cloud interface. Computing environments are private, secure, optimized for data transmission, and can be connected to build parallel architectures. Nevertheless, the performance is influenced by the latency and quality of the network and data center performance. The Google Cloud Robotics Platform, Bisong (2019), aims to leverage artificial intelligence, cloud computing, and robotics to provide utility services to customers. Amazon’s AWS RoboMaker, as reported in Chen and Luca (2021), allows users to develop code in the cloud, test it in the open-source robot simulator Gazebo, and then deploy updates directly to their robots running on the robotic operating system (ROS): unfortunately, this infrastructure does not allow for relocation of resources dynamically from the local execution to the cloud, but rather focuses more on fast prototyping and deployment of robotics application. The Honda RaaS platform (Nishimiya and Imai, 2021) aims to offer a wide range of robot and cloud-based services to support communication, robotic cooperation, and data sharing.

However, despite these efforts to standardize the cloud robotics architecture, the state-of-the-art in the field still needs to provide a software architecture capable of mixing the cloud and local computation with seamless relocation of skills based on the required performance and the network condition. As reported in Afrin et al. (2021), most of the available cloud architectures conduct limited evaluation and hardly ensure low latency data flow between robot and cloud. Furthermore, these frameworks are applications specific (Wan et al., 2018; Ma et al., 2015; Beigi et al., 2017), their in-built software systems are not always adaptable to decentralized resources (Ng et al., 2015), and they often fail to deal with the real-world environmental constraints [i.e., demonstrated only in simulation as in Berenson et al. (2012)] linked with the network conditions during local-to-cloud interaction.

Starting from this observation and exploiting our previous work on XBotCloud, described in Muratore et al. (2018), we propose a novel cloud robotics framework called XBot2D, which leverages the XBot2 software middleware (Laurenzi et al., 2023) and ROS to provide a hybrid cloud architecture capable of handling the on-the-fly flexible execution and relocation of robotic skills on the local system or the cloud based on the required latency, computation needs, and the current network quality, as well as the energy available during the execution of a mission. The proposed architecture considers the scalability of the approach employed and the security during the commission of the task to perform on the robotic system.

## 2 XBot2D hybrid cloud architecture

**FIGURE 2:**
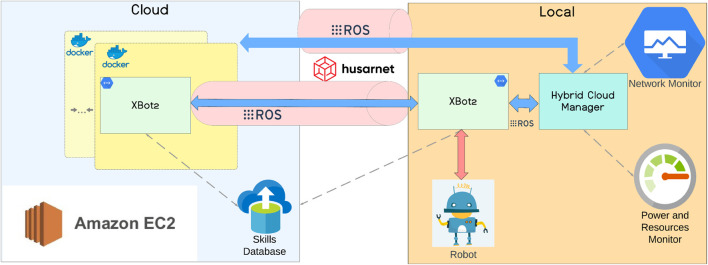
The XBot2D cloud robotics architecture following the RAaaS paradigm: the cloud and local dimensions (2D) and the main components of the framework are shown.

The XBot2D software architecture takes its name from the XBot2 middleware and the two-dimensional (2D) execution represented by the local robot and the cloud. Both aspects are critical for designing and implementing the proposed novel hybrid cloud framework and for realizing the transparent relocation of the robotics skills based on the system performances and the required execution profile. As can be seen from the overview of XBot2D reported in Figure 2, the main components realizing the proposed RAaaS system and the contributions of this work are:

• the XBot2 instances, which are capable of co-existing and acting in a transparent way, either from a docker container in the cloud or in the Local robotic system,
• the AWS EC2^1^ infrastructure and the Husarnet Peer-to-Peer VPN^2^, both capable of handling the ROS traffic in a scalable and secure way,
• the Hybrid Cloud Manager component, which takes into account the network and the power and resources status thanks to two monitors running locally, to communicate with the local or cloud XBot2 instances about the allocation of the dynamic skills, which are all stored in a Skills Database on the cloud.

### 2.1 XBot2 instances co-existence

XBot2^3^ is a novel RT middleware for robotic applications with a strong focus on modularity and reusability of components and seamless support for multi-threaded, mixed real-time (RT), and non-RT architecture. One of the main features of this framework is represented by a fully dynamic hardware abstraction layer (HAL), with support for on-the-fly device auto-discovery and the ability to generate high-level Application Programming Interfaces (APIs) for more transparent integration with the user’s custom code. One of the significant limitations of the current framework version is the lack of support for the transparent co-existence of multiple XBot2 instances controlling the same robotic system. To tackle the above issues, in this work, we exploited the XBot2 HAL flexibility to implement a ROS-based Device Driver and Client to enable multiple XBot2 instances co-existence. Given that in XBot2 a ROS built-in communication is available and the user code implementing the robotic skills is contained in control plugin objects, we decided to design a HAL Device capable of receiving the ROS status from the robot and sending the references through a ROS command message, exposing a high-level API for the plugins which is the same one exposed by a HAL responsible for the low-level communication with the robot (e.g., through EtherCAT drivers). In this way, the same plugin can be used without any code changes, either with an XBot2 instance directly connected with a HAL controlling the actual robot or with another instance of the XBot2 connected through ROS on the former instance.

**FIGURE 3:**
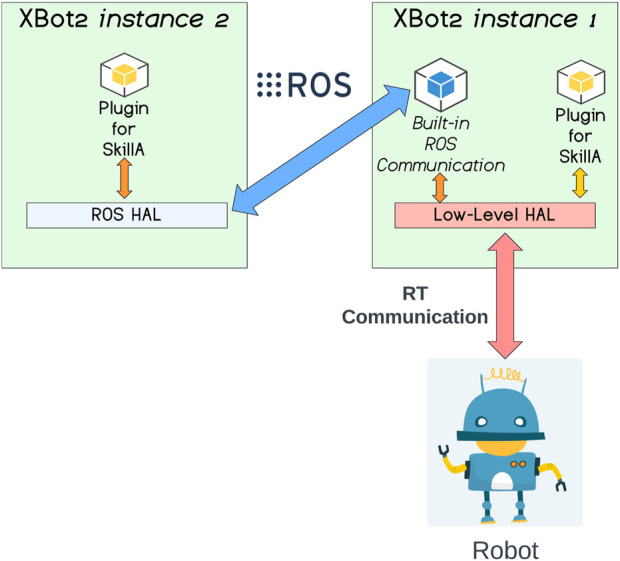
The XBot2 instances co-existence scheme employed in the XBot2D cloud architecture.

The diagram in Figure 3 explains this concept visually. Taking into account the implementation of a certain robotic skill (SkillA) with a XBot2 plugin, it can be observed that with no code changes, SkillA can execute on the XBot2 instance 1 directly on top of the Low-Level HAL, which performs an RT communication with the robot. At the same time the SkillA can run the XBot2 instance 2 passing trough the ROS Hal, the ROS communication channel with a set of topics, the built-in ROS communication mechanism implemented in the XBot2 and then again on the Low-Level HAL layer to communicate with the robot.

This mechanism is completely transparent to the user of the XBot2 and can be replicated in other instances of the framework.

### 2.2 Cloud infrastructure

As for its predecessor XBotCloud, XBot2D uses the Amazon Web Services Elastic Compute Cloud (AWS EC2) service to provide a secure and scalable RAaaS infrastructure. AWS EC2 was confirmed in the design phase of the XBot2D since, compared to other available solutions in the market, it offers a wider range of instance types (e.g., compute-optimized, memory-optimized, storage-optimized, and GPU instances), multiple layers of security, elastic IP addresses, and detailed monitoring and metrics for the instances in use.

For the Virtual Private Network (VPN) instead, we decided to go for Husarnet instead of the OpenVPN^4^ used in XBotCloud: this choice is justified by the low communication overhead introduced by Husarnet compared to other available VPNs, the built-in support for ROS and ROS2, and the peer-to-peer connection over the internet to ensure the lowest possible latency.

On top of this, in the cloud dimension, we built up a Docker architecture capable of working with isolated containers for the distinct XBot2 instances in use for different robotic systems: this represents a step-change for the portability and the deployment of the XBot2D into real-world ready settings.

### 2.3 Hybrid cloud manager

The hybrid cloud manager is the core layer of the XBot2D architecture since it coordinates and manages the XBot2 instances running both on the cloud and locally. The main entity handled by this component is called Skill and represents a possible robotics application (e.g., joint space or cartesian control, navigation, object recognition, etc.) implemented as an XBot2 plugin. The execution of the plugin in the system is organized according to a well-defined life cycle, implemented through a finite state machine controlled by a ROS service. When the user defines a particular mission, the necessary Skills are retrieved by the XBot2 instances, upon request from the hybrid cloud manager, inside the Skills database in the cloud. A Skill is categorized in terms of latency (latency-sensitive or latency-tolerant) and computational requirements: based on this, it will be executed and allocated in the local or cloud dimension through the above-described ROS service by the hybrid cloud manager.

Moreover, we added two components responsible for monitoring the network status, the power consumption, and the robot’s computational resources: they are in direct communication with the hybrid cloud manager, which can take actions depending on the performance required by the task to execute and the battery level of the robot. The network status indication can cause the offload of the Skill or the centralization on the local robot. All of the above has been designed to be transparent for the users and to introduce minimal overhead in terms of communication and performance: local RT Skills and cloud computational-intensive Skills can shift dimensions based on the needs calculated by the hybrid cloud manager. The current implementation of this component was designed to give flexibility to the users, and in this work, the validation carried out helped us understand how to tune the parameters of this layer based on the computational load and especially the network condition during the mission execution.

## 3 Experiments and results

Two sets of experiments were carried out to demonstrate the effectiveness of the proposed hybrid cloud architecture. We performed the validation on the CENTAURO robot, a dual arm robot equipped with a hybrid leg-wheel mobility system Kashiri et al. (2019). The experiments were executed both on the Gazebo simulation and on the real robot. In all the tests, we utilized actual cloud conditions with real-world network delay between our lab in Genova (Italy) and the cloud server allocated by AWS EC2 in Ireland. On top of this, we implemented a degrading network tool, built on top of the Traffic Control suite in the Linux kernel^5^ to simulate latency and packet loss typical of field robotics application scenarios. During all the experiments, the local execution on the RT layer is working at 1 *kHz* update frequency, while the communication with the cloud is handled at 200*Hz*.

The video of the main experiments carried out for the XBot2D hybrid cloud architecture can be found at this link https://youtu.be/uf7UAH4ebHM.

### 3.1 Single skill

**FIGURE 4:**
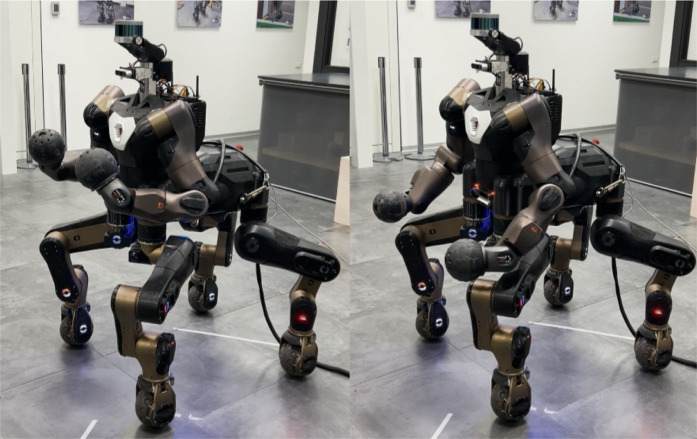
First set of experiment with the CENTAURO executing a single Skill to repetitively move its arms from an initial pose on the left to the one on the right.

**FIGURE 5:**
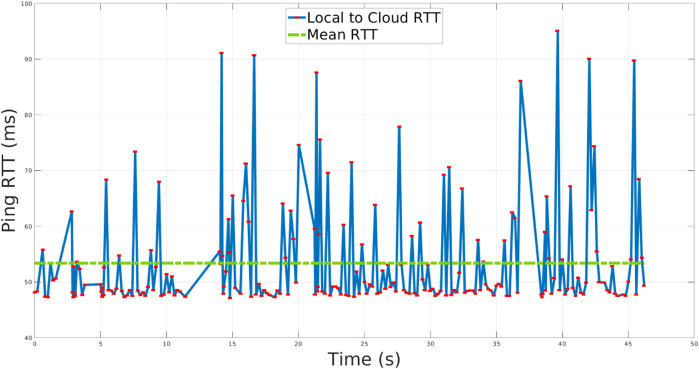
The RTT experienced from the Local to the Cloud during the validation of the XBot2D framework.

In the first set of tests a single Skill use-case is taken into account: the robot needs to perform a repetitive trajectory in the joint space from a certain position to another, as shown in Figure 4, in 5 s. During the execution, the Skill is firstly run on the Cloud and then transparently and dynamically ported on the local execution. The standard network conditions experienced in this validation set and also in the rest of the experiments are similar (since all of them were carried out in the same day): in Figure 5, we report a representative Local to Cloud Round Trip Time (RTT) graph built using the ping network utility retrieved through ROS from the network monitor running in the XBot2D. In the context of computer networking and telecommunications, the RTT is the time it takes for a packet of data to travel from a source to a destination and then back to the source. It is worth noticing that the mean value of the RTT during the experimental phase is around 53 *ms*, as shown in Figure 5 with a green dotted line.

**FIGURE 6:**
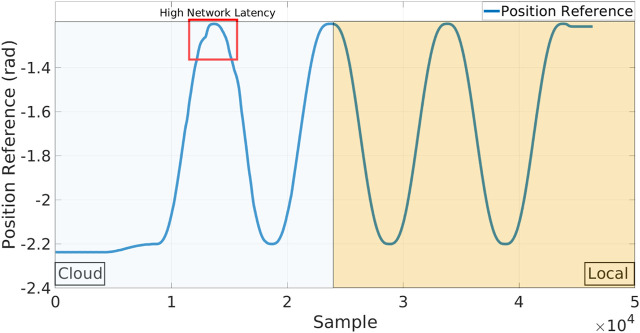
Plot of the position reference sent to the CENTAURO robot during the execution of the repetitive joint space control Skill. On the first part of the experiment (left, light blue background), the control is happening on the Cloud, while in the second part (right, light orange), it is working locally.

**FIGURE 7:**
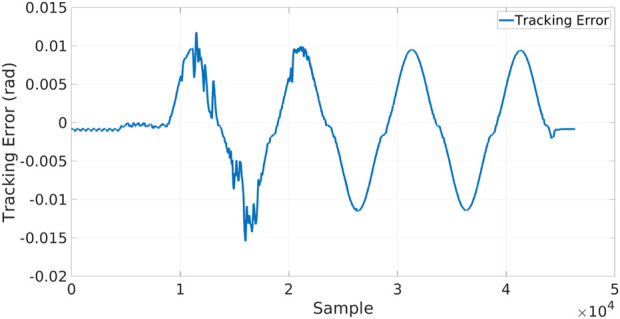
Tracking error between the reference and the motor position of the elbow joint during the single Skill validation.

In the Local XBot2 instance, the Skill is running inside the Xenomai RT Kernel patch^6^, assuring low-latency and predictable response time; at the same time the identical implementation is transparently executed on the cloud in non-RT mode. Indeed, thanks to the Skills Database, the hybrid cloud manager can enforce the XBot2 instances to use the same plugin implementation either in RT mode (if supported) or in the non-RT one. The position references commanded to the CENTAURO elbow joint are shown in the graph of Figure 6. During the first part of the experiment, the execution is happening on the Cloud, while in the second part, it is operating locally. It can be observed that the reallocation from the Cloud to the Local dimension is transparent, and XBot2D does not introduce any overhead on top of the possible network delay. A high network latency is indicated in the red box; during that time, as it can be observed, a not continuous position reference was fed to the robot from the cloud. In the plot in Figure 7, instead, it is reported the tracking error (i.e., the discrepancy between the position reference and the motor position) of the elbow joint during the experiment: the profile of this error clearly shows the difference between the cloud (on the first part of the samples) and the local (on the second part of the samples) executions.

**FIGURE 8:**
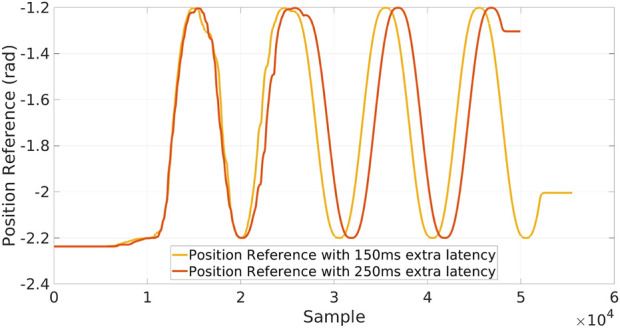
Single skill validation test with degraded network condition: in the first part of the experiment, the Skill is executed on the cloud and then relocated in the local dimension.

To understand the limitations of our software relocation strategy, we stressed the network condition during the execution of XBot2D: Figure 8 shows the same test as above, emulating a delay in the network respectively of 150 *ms*, and 250 *ms* on top of the standard RTT. It can be observed that the discontinuities in the first part of the execution on the Cloud can deteriorate the performance of the executed Skill on the robot and that the higher the delay, the larger the execution shifts in time.

### 3.2 Multiple skills with perception

In the second part of our validation, a set of two skills with different requirements have been taken into account:

1. A latency-sensitive mobility Skill commanding the wheels of the CENTAURO to go forward with a fixed velocity.
2. A computation-intensive gesture recognition Skill built on top of the mediapipe framework to command the stop of the mobility of the robot.

Given the above, we exploited the Intel RealSense D435i mounted on the head of CENTAURO to retrieve a compressed image streaming to feed the gesture recognition Skill. The full Husarnet VPN (with IPv6 addresses of the different hosts) is shown in Figure 10 on the right.

**FIGURE 9:**
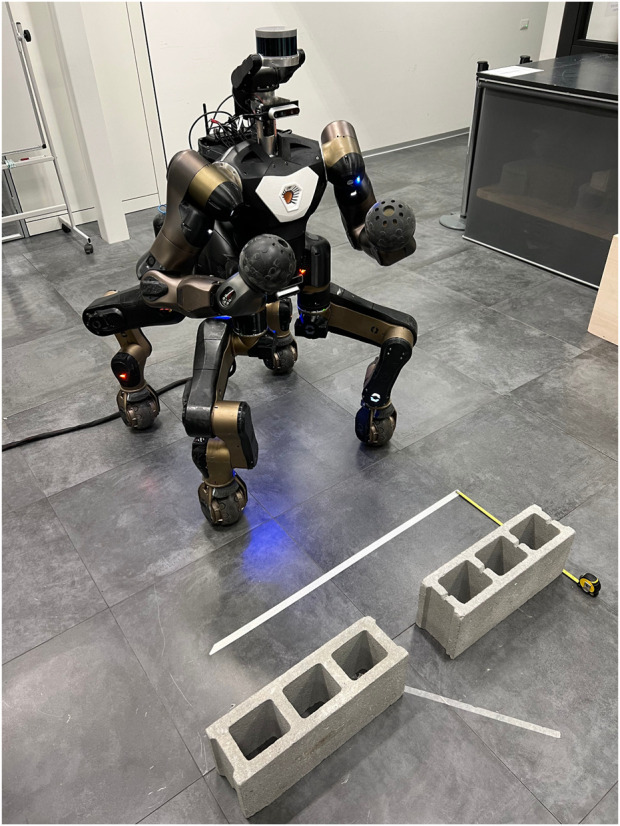
Scenario of the second set of experiments for the XBot2D validation.

**FIGURE 10:**
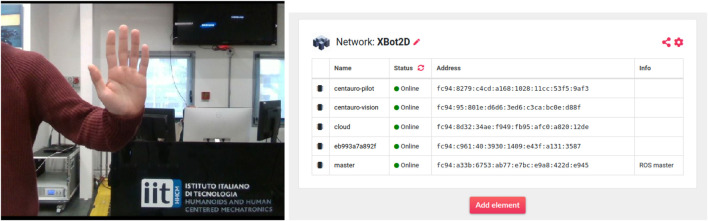
On the left, the “Stop” gesture performed by the operator; on the right the Husarnet network in use during the second set of experiments.

The scenario of this set of experiment is depicted in Figure 9: the CENTAURO robot is placed in front of two brick obstacles, and an operator is required to perform the gesture linked with the stop (open palm, as shown in Figure 10 on the left) as soon as the robot crosses the white line located at 32 *cm* from the obstacles. The online and closed-loop features of this mission are helpful in highlighting how the hybrid cloud manager can allocate the Skills on the two Dimensions.

As in the last part of the first set of experiments, we degraded the network on purpose to push the limits of the XBot2D architecture. We evaluated the robot’s reaction time during the execution of the two Skills in terms of distance error to the ideal stop precisely on the white line. In case the CENTAURO hits the obstacles, the experiment has been considered failed. Five different execution use-cases are considered and presented below:

1. both Skills are executed on the Local dimension,
2. the mobility Skill is executed locally, while the gesture recognition Skill is executed on the cloud,
3. the gesture recognition Skill is executed locally, while the mobility Skill is executed on the cloud,
4. both the Skills are executed on the Cloud dimension,
5. the mobility Skill is executed locally, while the gesture recognition Skill is firstly executed on the cloud and then, after a sever network degradation, dynamically reallocated on the Local dimension.

In Table 1, the results of the measured stop error are reported: it can be seen that a critical Skill to execute on the cloud is the gesture one, which has the most critical computational and communication requirements.

**TABLE 1:** Performance of the first four use-cases in terms of stop distance error.

Use-case	Stop distance error [cm] (with no extra delay)	Stop distance error [cm] (with 50 *ms* delay)	Stop distance error [cm] (with 150 *ms* delay)	Stop distance error [cm] (with 250 *ms* delay)
Gesture local - mobility local	1 ± 0.5	2 ± 0.5	1 ± 0.5	1 ± 0.5
Gesture local - mobility cloud	8.8 ± 1.5	13.3 ± 1.6	21.4 ± 2.3	FAILED
Mobility local - gesture cloud	15.4 ± 1.9	16.4 ± 1.7	25.8 ± 1.5	FAILED
Gesture cloud - mobility cloud	15.5 ± 1.3	19.3 ± 1.5	FAILED	FAILED

The precision of our measurement system is around 1 *cm*.

**FIGURE 11:**
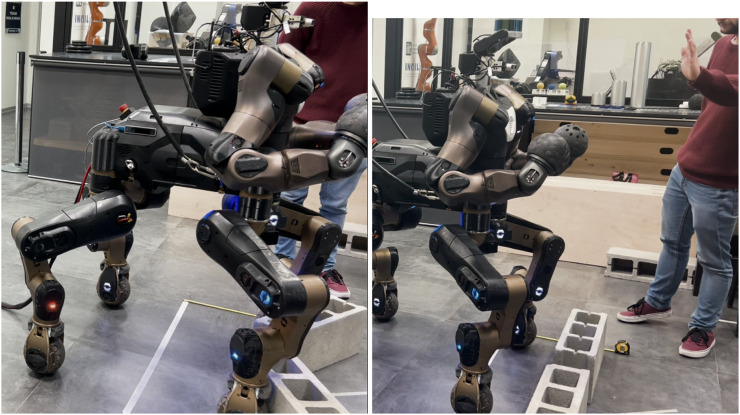
On the left cloud execution of the two skills of the second experimental set with a failure to stop the robot before the obstacles. On the right a successful trial with the XBot2D framework, capable of dynamically relocating the gesture recognition Skill from the cloud to the local dimension when the network degradation occurs.

Below the 250 *ms* delay we are able to succeed in all the use-cases (with different accuracy) except for the execution of both Skills on the Cloud. Starting from this observation, we decided to introduce also a packet loss emulation on top of the delay in our network degradation tools. The results in terms of execution from the cloud were poor, as it can be seen in Figure 11.

Given this, we enabled the hybrid cloud manager to offload the gesture recognition Skill on the Cloud only under good network conditions, otherwise an immediate relocation on the Local Dimension would be needed. The outcome of this kind of hybrid execution lead to the completion of the task: the degradation of the network with 150 *ms* delay and 20% packet loss makes the hybrid cloud manager reacting and reallocating the gesture Skill locally, demonstrating an distance stop error of 17.2*cm*, as can be observed in Figure 11 on the right.

## 4 Conclusion and future works

In this work, we presented a novel hybrid cloud robotics architecture called XBot2D, implementing a RAaaS model to provide users with a scalable and secure infrastructure capable of overcoming the limitation of communication constraints in field robotics applications. Indeed, thanks to a bi-dimensional approach (2D), a set of robotics Skills can be executed and relocated either on the cloud or the local robotic system in a completely transparent fashion, assuring resilience against communication latency and degradation. Moreover, the XBot2D framework has been validated in a set of realistic tasks involving the CENTAURO robot developed at IIT employing a real cloud infrastructure, including an AWS EC2 instance to provide support for demanding computational skills and the Husarnet VPN, which assures low latency ad secure communication over the internet. The hybrid cloud architecture demonstrated the ability to reallocate Skills dynamically without adding overhead on top of the network round trip time between the robot and the cloud servers. In this way, the framework can provide seamless support for latency-sensitive Skills, assuring RT execution locally and A.I.-based Skills, which require high computational resources of the cloud, effectively minimizing the energy consumption on the robotic system. The preliminary results reported in this paper are crucial to obtain a real-world ready cloud architecture that can demonstrate to work outside the lab environment.

Our goal is to extend the XBot2D, adding the third dimension of the edge computing infrastructure: thanks to it, the Skills that require moderate latency can execute closer to the robot, reducing the possible communication delay with the cloud. Another future direction relies upon integrating the ROS2 communication framework to leverage the DDS approach and avoid multi-master strategies to reduce the bandwidth used by the ROS ecosystem. As a final step, we intend to support heterogeneous multi-robot systems inside the XBot2D framework to realize tasks and execute missions by exploiting the cooperation of robots with different skills and capabilities.

## Data Availability

The raw data supporting the conclusion of this article will be made available by the authors, without undue reservation.

## Footnotes

1
https://aws.amazon.com/ec2/?nc1=h_ls

2
https://husarnet.com/

3
https://advrhumanoids.github.io/xbot2/

4
https://openvpn.net/

5
https://man7.org/linux/man-pages/man8/tc.8.html

6
https://source.denx.de/Xenomai/xenomai
